# Controlling signal transport in a carbon nanotube opto-transistor

**DOI:** 10.1038/srep37193

**Published:** 2016-11-16

**Authors:** Jinjin Li, Yanhui Chu, Ka-Di Zhu

**Affiliations:** 1 Key laboratory for Thin Film and Microfabrication of Ministry of Education, Department of Micro/Nano-electronics, Shanghai Jiao Tong University, 800 Dong Chuan Road, Shanghai 200240, P. R. China; 2 School of Materials Science and Engineering, South China University of Technology, 381 Wushan Road, Tianhe District, Guangzhou, 510641, P. R. China; 3 Department of Physics and Astronomy, Shanghai Jiao Tong University, 800 Dong Chuan Road, Shanghai 200240, P. R. China

## Abstract

With the highly competitive development of communication technologies, modern information manufactures place high importance on the ability to control the transmitted signal using easy miniaturization materials. A controlled and miniaturized optical information device is, therefore, vital for researchers in information and communication fields. Here we propose a controlled signal transport in a doubly clamped carbon nanotube system, where the transmitted signal can be controlled by another pump beam. Pump off results in the transmitted signal off, while pump on results in the transmitted signal on. The more pump, the more amplified output signal transmission. Analogous with traditional cavity optomechanical system, the role of optical cavity is played by a localized exciton in carbon nanotube while the role of the mechanical element is played by the nanotube vibrations, which enables the realization of an opto-transistor based on carbon nanotube. Since the signal amplification and attenuation have been observed in traditional optomechanical system, and the nanotube optomechanical system has been realized in laboratory, the proposed carbon nanotube opto-transistor could be implemented in current experiments and open the door to potential applications in modern optical networks and future quantum networks.

An optical cavity coupled to a mechanical oscillator via radiation pressure is generally called a cavity optomechanical system, which has been appointed as the boundary between classical and quantum mechanical systems^1^,^2^,^3^. Cavity optomechanical system naturally offers a method for both detecting mechanical motion and cooling a mechanical mode to its ground state. In general, the fundamental researches have always focused on measurement and control of the mechanical oscillator while coupling with external optical fields via radiation pressure^4^,^5^. However, the mechanical oscillator can also affect the dynamics of cavity field. Therefore, recent experiments have reached a regime where the back-action of photons caused by the interaction between the radiation pressure and the mechanical oscillator influences the optomechanical dynamics significantly, producing plenty of long-anticipated phenomena, such as optomechanically induced transparency (OMIT)^6^,^7^ and amplification (OMIA)^8^, great change of the group velocity of light^9^, and mass sensitive to the external particles^10^.

One of the further development in cavity optomechanical system concentrates on the substitution of mechanical element or optical cavity to other nanometer or micrometer scale systems, which not only inherits the properties of cavity optomechanics but also develops advantages of new materials with new functions^10^. With this background, optomechanical systems with dielectric membrane^11^, multilayer graphene^12^, silicon thin film^9^, atomic gas^13^ *et al*. continuously spring up to implement more advanced features. For example, some atomic gas or fermion gas combined cavity quantum electrodynamics (QED) has been used to realize the cavity optomechanical system. Brennecke *et al*.^13^ have experimentally reported a cavity optomechanical system in which a collective density excitation of a Bose-Einstein condensate serves as the mechanical oscillator coupled to the cavity field, which brought the merits of Bose-Einstein condensate into cavity optomechanical system and realized the quantum phase related nonlocal coupling in the first time. Recently, Hümmer *et al*.^14^ have realized the optomechanical system experimentally in a hybrid cavity-nanotube system, where the coupling between photons in cavity and the vibrations of carbon nanotube resulted in the enhanced optical spectrum, which could be used for gaining quantum control in nanotube materials. Other experimental work regarding to carbon nanotube based optomechanical system can be found in refs 15 and 16. Such nanotube based optomechanical systems, concentrating the advantages of carbon nanotube and cavity optomechanical system, provided a platfrom for nanotube based quantum control and quantum information.

Carbon nanotube (CNT) is one of the most potential candidate for various sensing technology applications^17^,^18^, due to their outstanding characteristics, including ultralight weight, strong nonlinear response, long vibration lifetime, easy fabrication and weak dissipation, which enables CNT based ultrasensitive photonic sensors such as photovoltaics, nanotherapeutics, bioimaging, and photocatalysis^19^,^20^,^21^,^22^. Another merit for CNT is the insensitivity to external temperature and perturbation, which will enhance the transmitted signal carried by CNT and reduce the noise. In this article, we demonstrate that in the presence of a strong pump field and a weak signal field, a suspended CNT resonator can be treated as an optomechanical system. Compared with the optomechanical system implemented by Hümmer *et al*., where a carbon nanotube was coupled to an optical cavity, the role of optical cavity in our scheme is played by the localized exciton. Such localized exciton can be formed in the center of carbon nanotube by applying a transversal inhomogenous electric field via CNT. The electric field induced exciton in CNT, possessing the same two-level structure as an optical cavity (a ground state and a first excited state), confines electrons in a small region and provides a static accommodation for photos-phonons interference. The usage of a localized exciton to substitute an optical cavity in CNT optomechanical system, for the most part that avoids the photo-photo interference, provides a simpler hybrid research structure and would be easily achieved in experiment. The signal transmission spectrum indicates that the CNT optomechanical system can realize optomechanically induced transparency (OMIT)^6^ and amplification(OMIA)^8^,^23^, as well as electromagnetically induced absorption (EIA)^24^ simultaneously, which can be used to devise a quantum optomechanical transistor where the transmitted signal through CNT can be controlled by the second strong pump filed. Turning on and off the incident pump laser result in the amplification and the attenuation of the signal laser, respectively. Since the nanotube based optomechanical system was realized by Hümmer *et al*. and the OMIT, OMIA, and EIA effects were achieved by Shen *et al*. experimentally in optomechanical system, the current state of the work presented in this manuscript can be implemented in laboratory.

## Theory

**Figure 1:**
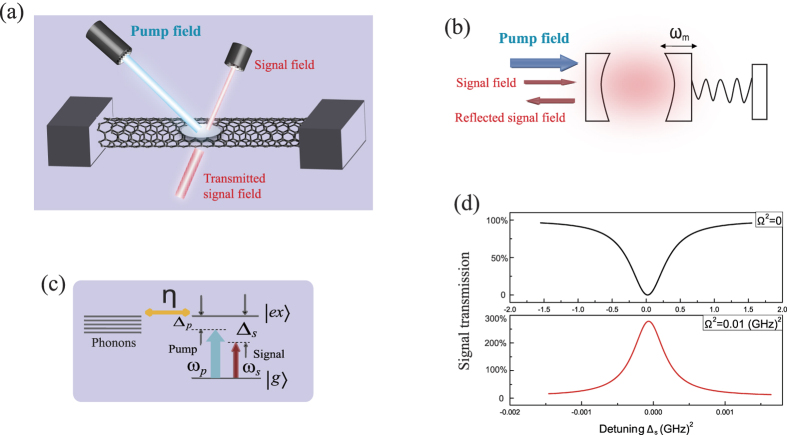
(**a**) Schematic of a suspended carbon nanotube resonator with a localized exciton in the middle, in the presence of a strong pump laser and a weak signal laser. The vibrational modes of nanotube resonator can be treated as phonon modes. (**b**) Typical cavity optomechanical system. The left mirror is fixed, while the right movable mirror couples to the optical cavity field via radiation pressure. (**c**) The energy levels of localized exciton in carbon nanotube. The photons-phonons coupling via radiation pressure in conventional optomechanical system is in analogy with the exciton-phonons coupling via deformation potential in suspended carbon nanotube. (**d**) Attenuation (pump off) and amplification (pump on) of the signal laser, which correspond to the switching and amplification behaviors in CNT. The parameters used are (*ω*_*n*_/2*π*, Γ_2_, *γ*_*n*_, Δ_*p*_) = (725, 310, 0.8, −725) MHz, *η* = 0.17.

We consider a suspended carbon nanotube in the presence of a strong pump laser (with frequency *ω*_*p*_) and a weak signal laser (with frequency *ω*_*s*_) as shown in Fig. 1(a). Figure 1(b) shows the traditional cavity optomechanical system, where the left mirror is fixed and the right movable mirror couples to the optical cavity field via radiation pressure. The comparisons between carbon nanotube based optomechanical system and traditional cavity optomechanical system are shown in Fig. 1(a) and (b). The structure of a semiconducting carbon nanotube can be viewed as a graphene rolled into a cylinder, where the mass of exciton is localized in the center of CNT via the spatial modulation of the Stark-shift arising from a static inhomogenous electric field. In this case, the carbon nanotube acts like a quantum dot (QD) where electrons are confined to a small region of carbon nanotube. This localized QD is formed in the segment of nanotube between doubly clamped suspensions, leading to a quantized energy spectrum in the longitudinal direction^25^. The roles of localized exciton and vibrational mode of carbon nanotube in Fig. 1(a) can be treated as optical cavity and mechanical motion in traditional optomechanical system in Fig. 1(b).

Figure 1(c) shows the energy levels of localized exciton and mechanical motion of carbon nanotube when applying a strong pump field and a weak signal field in CNT system. The localized exciton can be modeled as a two-level structure consisting of the ground state |*g*〉 and the first excited state (single exciton) |*ex*〉. Such an exciton can be characterized by the pseudospin −1/2 operators *S*^±^ and *S*^*z*^. When dressing with the mechanical motion of CNT (the mechanical motion can be treated as phonon mode^26^), the energy level of exciton exhibits metastable type characteristic, which has the same feature as traditional cavity optomechanical system^6^,^27^. In this case, the photon-phonon coupling via radiation pressure in traditional optomechanical system is in analogy with the exciton-phonon coupling via deformation potential in suspended carbon nanotube. Pirkkalainen and co-workers^28^ have demonstrated experimentally that a two-level system can be treated as an optomechanical system when coupling with a mechanical motion, where the mechanical motion provides an extra energy level for the two-level structure, enables the optical interference and strongly enhances the optical spectrum. Figure 1(d), which will be discussed in the following sections, shows the signal transmissions when pumping on (red curve) and pumping off (black curve) the pump field, which correspond to the the signal absorption and amplification, respectively.

The Hamiltonian of the localized two-level exciton can be described as *H*_*ex*_ = *ħω*_*ex*_*S*^*z*^, where *ω*_*ex*_ is the frequency of exciton. The lowest-energy resonance of CNT corresponds to the fundamental flexural mode with frequency *ω*_*n*_ and the resonator is assumed to be characterized by sufficiently high quality factors^25^. The eigenmode of CNT can be described by a quantum harmonic oscillator with *b* and *b*^+^ (the bosonic annihilation and creation operators with a quantum energy *ħω*_*n*_). Therefore, the vibrational Hamiltonian of CNT is given by *H*_*n*_ = *ħω*_*n*_*b*^+^*b*, where the vibration modes of CNT is treated as phonon modes. In the simultaneous presence of a strong pump field and a weak signal field, the Hamiltonian of the CNT system can be written as^25^,^26^,^29^:


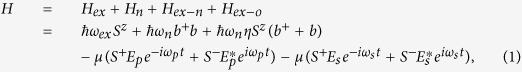


where 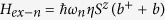 represents the interaction between the nanotube resonator and the exciton^25^,^30^, *η* is the coupling strength. 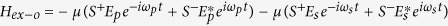 describes the coupling between exciton and two optical fields, where *E*_*p*_ and *E*_*s*_ are slowly varying envelopes of the pump field and the signal field, respectively, and *μ* is the electric dipole moment of the exciton. In a frame rotating at the pump field frequency *ω*_*p*_, the total Hamiltonian of the CNT system reads as follows:


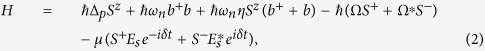


where Δ_*p*_ = *ω*_*ex*_ − *ω*_*p*_ is the detuning of pump field and exciton, *δ* = *ω*_*s*_ − *ω*_*p*_ is the detuning of signal field and pump field, and Ω = *μE*_*p*_/*ħ* is the Rabi frequency of the pump field.

In the following, we consider the decoherence and relaxation of exciton and CNT resonance mode in combination with their interaction to external environments into the Hamiltonian^31^,^32^,^33^,^34^. We use the independent ensembles of harmonic oscillators with spectral densities to describe the environments, where CNT interacts bilinearly with external environment via its position operators, and exciton interacts with environment through *S*^*x*^ operator and *S*^*z*^ operator. The *S*^*x*^ coupling to the environment indicates the relaxation process of exciton, while the *S*^*z*^ coupling to the environment indicates the pure dephasing process of exciton^31^,^32^,^33^,^34^. Since 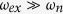, it is reasonable that the exciton-environment coupling can be treated in the rotating-wave approximation, whereas the CNT-environment coupling can not use the same treatment.

Tracing out the environmental degrees of freedom in terms of the standard procedure^31^,^32^,^33^,^34^, we can obtain the Born-Markovian master equation of the reduced density matrix of the coupled system *ρ*(*t*) as


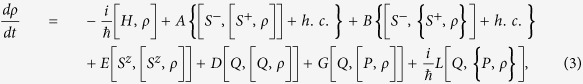


where *Q* = −*η*(*b*^+^ + *b*) and *P* are the position and the momentum operators of CNT, respectively. The coefficients A, B, E, D, G and L correspond to the characteristics of the coupling, and to the structure and properties of the environments. Their explicit forms can be written as:


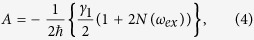



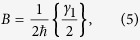



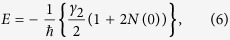



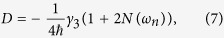



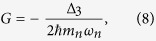



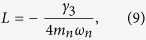


where *γ*_1_ = 2*πJ*_*x*_(*ω*_*ex*_), *γ*_2_ = 2*πJ*_*z*_(0), *γ*_3_ = 2*πJ*_*c*_(*ω*_*n*_), 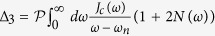. 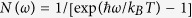 is the Bose-Einstein distribution of the thermal equilibrium environments; 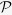 denotes the principal value of the argument; *m*_*n*_ is the effective mass of CNT; *J*_*x*_, *J*_*z*_ and *J*_*c*_ describe the spectral densities of the respective environment coupled to the exciton through *S*^*x*^ and *S*^*z*^, and to CNT through *Q*, respectively.

According to the master equation (3), we can obtain the equation of motion for the expectation value of any physical operator *O* of the coupled system by calculating 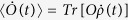. We thus have:


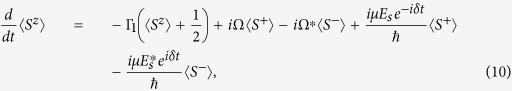



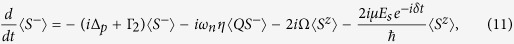



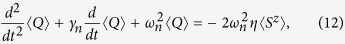


where Γ_1_ and Γ_2_ are the exciton relaxation rate and dephasing rate, respectively, *γ*_*n*_ is the decay rate of CNT^25^,^35^. They are derived microscopically as:


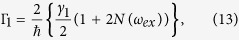



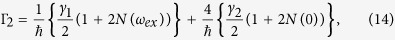



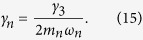


Obviously, when pure dephasing coupling is neglected, i.e., *γ*_2_ = 0, we can get Γ_1_ = 2Γ_2_. In order to solve these equations, we take the semiclassical approach by factorizing the nanotube and exciton degrees of freedom, i.e., 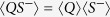, in which any entanglement between these systems should be ignored. And then we make the ansatz^26^: 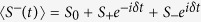, 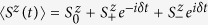, 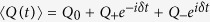. Upon substituting the approximations to Eqs (10)–(12), 36, 37 and working to the lowest order in *E*_*s*_, but to all orders in *E*_*p*_, we finally obtain the linear optical susceptibility *S*_+_ in the steady state as following:


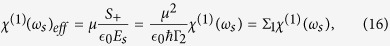


where 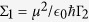 (*ϵ*_0_ is the dielectric constant of vacuum). The dimensionless linear optical susceptibility is given by


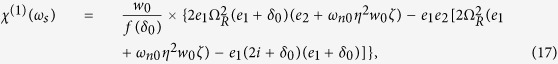


where 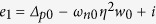, 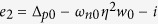, and 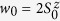, Γ_1_ = 2Γ_2_, 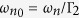, 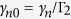, 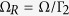, *δ*_0_ = *δ*/Γ_2_, Δ_*p*0_ = Δ_*p*_/Γ_2_.

The auxiliary function *ζ*(*δ*_0_) and function *f*(*δ*_0_) are given by


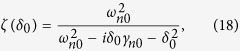



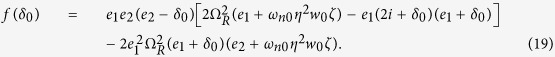


The population inversion *w*_0_ of the exciton is determined by the following equation


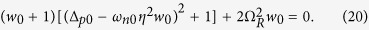


## Results and Discussion

**Figure 2:**
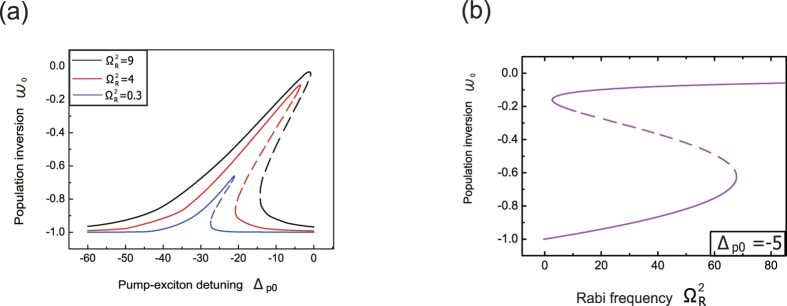
(**a**) Steady-state population inversion *w*_0_ as a function of pump-exciton detuning with different Rabi frequencies of pump field. The dashed curves correspond to the unstable state. (**b**) Population inversion *w*_0_ as a function of pump Rabi frequency. The parameters used are *η* = 0.8 and *ω*_*n*0_ = 50.

The cubic equation (20) is the characteristic feature of optical multistability^36^,^37^. Figure 2 shows the steady-state value of population inversion *w*_0_ as a function of pump-exciton detuning when fixing the pump Rabi frequency (Fig. 2(a)), and as a function of pump Rabi frequency when fixing the pump-exciton detuning (Fig. 2(b)). The dashed curves and the solid curves correspond to the unstable and bistable states, respectively. Actually, there are some published works demonstrated that a mechanical motion (i.e., carbon nanotube) coupled to a two-level structure (i.e., optical cavity or exciton) can be served as an optomechanical system^14^,^28^. But the bistable behavior shown in Fig. 2, a hallmark of optomechanical system, provides a further strong evidence that under the radiation of two optical fields, a suspended carbon nanotube acts as an optomechanical system^36^,^37^. In fact, we notice that the value of population inversion *w*_0_ is confined between 0 and −1, which is distinguish from the conventional cavity optomechanical system where the number of photons in cavity is determined by the incident pump laser. That is to say, the input pump field in conventional cavity optomechanical system directly influences the coupling between cavity photons and mechanical oscillator. However, in CNT based optomechanical system, the deformation coupling of exciton-phonon is independent with the pump field, because the population inversion of exciton in Fig. 2 can only take the value between 0 and −1 via the interaction between exciton and pump field.

A suspended carbon nanotube should exhibit optomechanical properties in optical fields, i.e., optomechanically induced transparency (OMIT)^6^,^7^ and amplification (OMIA)^8^, great change of the group velocity of light^9^ *et al*., since we have demonstrated its behavior of optomechanics. Figure 1(d) shows the switching behavior of CNT resonator under the radiation of two optical fields, where the signal transmission spectra are plotted as a function of frequency detuning between signal field and exciton with Δ_*s*_ = *ω*_*s*_ − *ω*_*ex*_. The calculated parameters in Fig. 1(d) are (*ω*_*n*_/2*π*, Γ_2_, *γ*_*n*_, *η*) = (725 *MHz*, 310 *MHz*, 0.8 *MHz*, 0.17)^25^, which are selected from a realistic CNT with an ambient temperature of 4.2 K and a quality factor of *Q* = 908. The top black curve indicates that when shelving the pump beam but only applying a signal beam, the suspended CNT attenuates the weak signal beam totally. This dip arises from the usual exciton absorption resonance. However, when turning on the pump field and fixing the pump-exciton detuning at Δ_*p*_ = −*ω*_*n*_, the dip switches to a transmission peak immediately (see the bottom red curve in Fig. 1(d)). The 300% signal transmission shows that when Ω^2^ = 0.01 (GHz)^2^, the strength of output signal is three times larger than incident signal. This amplification comes from the quantum interference between the CNT vibration modes (phonon modes) and the beat of the two optical fields via the quantum dot exciton. Figure 1(c) explains this amplified resonance process, where the two energy levels of exciton split into several metastable levels when dressing with the phonon modes, which allows the electrons making energy levels’ transitions between them when applying a strong pump field and eventually results in the constructive interference and amplified signal. The transparency window shown in the top curve of Fig. 1(d) comes from the interactions between excitonic resonance and CNT vibrations via deformation potential, which is analogy with the interactions between photons and mechanical resonator via radiation pressure in traditional optomechanical system^6^. We name the transparency window shown in CNT optomechanical system as “phonon induced transparency(PIT)” as a comparison with the optomechanically induced transparency(OMIT) window in traditional cavity optomechanical system proposed by Kippenberg *et al*.^6^ in laboratory.

**Figure 3:**
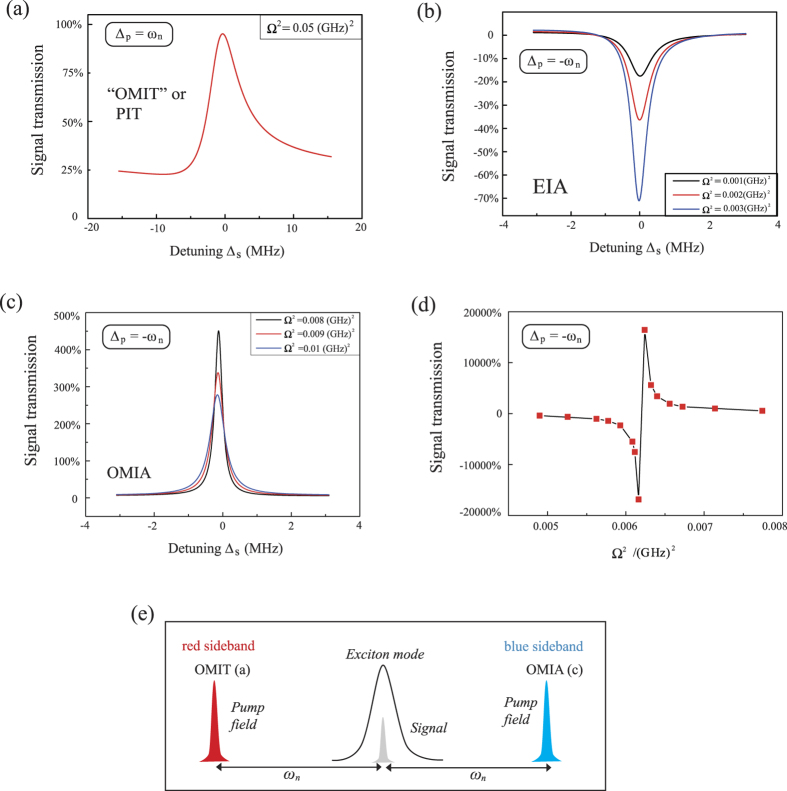
Plots of optomechanically induced transparency (OMIT) or phonon induced transparency (PIT), electromagnetically induced absorption (EIA) and optomechanically induced amplification (OMIA). Signal transmission as a function of signal-exciton detuning for (**a**) Δ_*p*_ = *ω*_*n*_. (**b**) Δ_*p*_ = −*ω*_*n*_ with small Rabi frequencies of pump field. (**c**) Δ_*p*_ = −*ω*_*n*_ with large Rabi frequencies of pump field. (**d**) The relationship between the signal transmission and the intensity of pump field. The other parameters used are the same as the ones in Fig. 1(d). (**e**) The OMIT and OMIA processes, which correspond to the red sideband (Δ_*p*_ = *ω*_*n*_) and blue sideband Δ_*p*_ = *ω*_*n*_, respectively.

Figure 3(a) exhibits the PIT effect in a suspended CNT system where the exciton is driven on its red sideband, i.e., Δ_*p*_ = *ω*_*n*_, which matches the experimental results obtained by Kippenberg *et al*. very well^6^. However, when switching the pump-exciton detuning to −*ω*_*ph*_, the output signal turns to an absorption spectrum in Fig. 3(b). From Ω^2^ = 0.001 (*GHz*)^2^ to 0.003 (*GHz*)^2^, the negative transmission of output signal increases with the increasing of pump intensity, which is the so-called electromagnetically induced absorption (EIA)^24^. If the pump field continues to increase, the negative signal transmission in Fig. 3(b) switches to positive transmission (Fig. 3(c)), which is the optomechanically induced amplification (OMIA)^8^,^23^. Recently, Shen and co-workers^8^ realized the OMIT and OMIA processes in optomechanical system at red sideband and blue sideband, respectively. In this case, the OMIT and OMIA effects, treated as other hallmarks of optomechanical system, are reasonable to be presented in the proposed CNT optomechanical system. Besides, the OMIA process in Fig. 3(c) exhibits the same characteristic with traditional optomechanical system, which generates an amplification from 300% to 460% as the pump increased from Ω^2^ = 0.008 (*GHz*)^2^ to 0.01 (*GHz*)^2 ^8,23. The signal transmission as a function of pump Rabi frequency is plotted in Fig. 3(d), which shows that a switching point from EIA to OMIA is around Ω^2^ = 0.0063 (GHz)^2^. It can be seen that the gain of signal laser can be 200 times larger than the incident signal at Ω^2^ = 0.0063 (GHz)^2^, which means that the optomechanical system with a suspended CNT can not only switches the signal from off to on, but also serves as a signal amplifier via exciton-phonons interactions.

Figure 3 shows us an important fact: when the intensity of pump field increases from 0 to a larger value, the transmission of signal laser will present evolutions from OMIT, EIA to OMIA, which provides an effective platform for the transmitted signal control. In addition, the switching and amplifying behaviors of doubly clamped CNT shown in Figs 1(d) and 3 demonstrates that in the region of Ω^2^ ≥ 0.0063 (GHz)^2^, the CNT optomechanical system acts as a quantum opto-transistor under the radiation of two optical fields. The pump laser, like a signal controller, regulates the amplification and attenuation of signal laser, while the CNT resonator behaves like a signal transport carrier. Figure 3(e) shows the two configurations of the pump field, where the red sideband corresponds to Δ_*p*_ = *ω*_*n*_ while the blue sideband corresponds to Δ_*p*_ = −*ω*_*n*_. The red sideband, an effective photon-phonon beam-splitter-like interaction, leads to coherent conversion and induces a transparent window (OMIT) (Fig. 3(a)); while the blue sideband, an effective photon-phonon pair generation process, leads to optomechanically induced amplification (OMIA) (Fig. 3(c))^8^.

## Conclusions

In summary, we have theoretically realized a transmitted signal control in a doubly clamped carbon nanotube system, which can be served as an opto-transistor via attenuation and amplification of signal by another pump field. The pump field, like a switch, directly controls the output signal; turning on and off the pump field correspond to amplification and attenuation of the signal field, respectively. The optical cavity coupling to mechanical oscillator modes in traditional optomechanical system is analogy with the exciton interacting with CNT vibration modes presented here, where the optomechanically induced transparency (OMIT) and amplification(OMIA)^8^,^23^, as well as electromagnetically induced absorption (EIA) are demonstrated to exist. According to the proposed CNT with localized exciton has been demonstrated as an optomechanical system; the experiment of CNT coupled to an optical cavity has been implemented in laboratory to realize the optical amplification; and the OMIT, OMIA processes have been achieved in optomechanical system experimentally, we believe that the OMIT, OMIA and EIA processes in CNT optomechanical system could be achieved in current experiments with the technique of optomechanics. For these reasons, the proposed CNT opto-transistor could open the door to CNT related potential applications, such as all-optical logic circuits and quantum repeaters.

## Additional Information

**How to cite this article**: Li, J. *et al*. Controlling signal transport in a carbon nanotube opto-transistor. *Sci. Rep.* **6**, 37193; doi: 10.1038/srep37193 (2016).

**Publisher’s note**: Springer Nature remains neutral with regard to jurisdictional claims in published maps and institutional affiliations.
